# A Two-Level Speaker Identification System via Fusion of Heterogeneous Classifiers and Complementary Feature Cooperation

**DOI:** 10.3390/s21155097

**Published:** 2021-07-28

**Authors:** Mohammad Al-Qaderi, Elfituri Lahamer, Ahmad Rad

**Affiliations:** 1 Department of Mechatronics Engineering, Faculty of Engineering, The Hashemite University, P.O. Box 330127, Zarqa 13133, Jordan; mohammadk_al@hu.edu.jo; 2 Autonomous and Intelligent Systems Laboratory, School of Mechatronic Systems Engineering, Simon Fraser University, Surrey, BC V3T 0A3, Canada; elahemer@sfu.ca

**Keywords:** speaker recognition system, limited speech data, short utterances, social robots, social human-robot interaction, two-stage classifier, fuzzy fusion

## Abstract

We present a new architecture to address the challenges of speaker identification that arise in interaction of humans with social robots. Though deep learning systems have led to impressive performance in many speech applications, limited speech data at training stage and short utterances with background noise at test stage present challenges and are still open problems as no optimum solution has been reported to date. The proposed design employs a generative model namely the Gaussian mixture model (GMM) and a discriminative model—support vector machine (SVM) classifiers as well as prosodic features and short-term spectral features to concurrently classify a speaker’s gender and his/her identity. The proposed architecture works in a semi-sequential manner consisting of two stages: the first classifier exploits the prosodic features to determine the speaker’s gender which in turn is used with the short-term spectral features as inputs to the second classifier system in order to identify the speaker. The second classifier system employs two types of short-term spectral features; namely mel-frequency cepstral coefficients (MFCC) and gammatone frequency cepstral coefficients (GFCC) as well as gender information as inputs to two different classifiers (GMM and GMM supervector-based SVM) which in total leads to construction of four classifiers. The outputs from the second stage classifiers; namely GMM-MFCC maximum likelihood classifier (MLC), GMM-GFCC MLC, GMM-MFCC supervector SVM, and GMM-GFCC supervector SVM are fused at score level by the weighted Borda count approach. The weight factors are computed on the fly via Mamdani fuzzy inference system that its inputs are the signal to noise ratio and the length of utterance. Experimental evaluations suggest that the proposed architecture and the fusion framework are promising and can improve the recognition performance of the system in challenging environments where the signal-to-noise ratio is low, and the length of utterance is short; such scenarios often arise in social robot interactions with humans.

## 1. Introduction

Voice modality is central to most creatures, particularly in human interactions among themselves. It is envisaged that it is also sensible that social robots communicate with humans through voice. There are three related yet different challenges that researchers are currently engaged towards realization of voice as a medium through which robots and humans interact: Who is speaking? What is being said? Which language is being spoken? This paper is concerned with the first question. Humans are better in person recognition by face than voice [1]. However, in machines, voice modality provides unique features in biometric person identification systems as opposed to face, fingerprint, gait, or iris. The speech signal is captured dynamically over a period of few seconds; therefore, variation in speaker’s model can be monitored [2]. Another advantage of using speech signal is the availability of numerous devices such as microphones, cellphone, and soundcards that can be used to capture the speech signal [3]. Moreover, there are many scenarios of social human–robot interactions where all the above mentioned biometric modalities are not simultaneously available due to limitation of robot’s sensory system or the occlusion of target person by static or dynamic objects such as furniture and other persons who share the same environment with the social robot. These situations may arise in many social settings like homes, hospitals, airports, etc.

Speaker recognition systems can be classified into text-dependent speaker recognition and text-independent speaker recognition. The former system is employed when the speaker is cooperative and willing to pronounce fixed utterances as an authentication password or prompted by the system to pronounce pre-defined phrases that have been already registered in the system during the enrolment process. This scenario is readily used in speaker authentication systems where they extract signature(s) from a fixed utterance pronounced by an unknown speaker and verify if this utterance corresponds to the claimed identity. Even though the text-independent speaker recognition can be used as a speaker verification system, the system demonstrates its advantages in other applications where the speaker needs to be identified from unconstrained utterances as in the case of identifying a speaker by a social robot in a social human–robot interaction scenario. Constrained utterances used in text-dependent speaker recognition systems limit the functionality of the system in some applications such as social robot, speaker diarisation, intelligent answering machine with personalized caller greeting, and forensic investigation of telephone conversations. A speaker identification system for social human–robot interaction, which is the main motivation of this research, should be able to extract a voice signature from unconstrained utterances while maintains its performance at challenging scenarios including various low signal-to-noise ratio and short length of utterance as well as different types of noise. However, most of the state-of-the-art speaker recognition systems, including those based on deep learning algorithms, have achieved significant performance on verification tasks which are not suitable for social human–robot interaction [4,5,6]. In speaker diarisation, which is a key feature for social robots, also known as “who spoke when”, a speech signal is partitioned into homogenous segments according to speaker identity [7]. Hence, the speaker identity must be induced by extracting features from unconstrained utterances that may be short and not have been used in the training stage.

The background speaker model is used in speaker recognition literature extensively as a way to enhance the robustness and computational efficiency of speaker recognition system [3]. Unlike other biometrics (such as face, fingerprint, iris, and hand geometry), voice biometrics is prone to substantial variability. There are many factors contributing to this variability including but not limited to changes in vocal tract and how a speaker produces speech (intrinsic-based source of variations), variation of speech signal capturing and transmission (external-based source of variations), and variation in languages and dialects spoken that are used in conversation (conversation-based source of variation). In situations referred to as within-speaker variability, the same person does not utter the same words in the same way. These conditions could arise due to physiological reasons (e.g., illness, being intoxicated, or aging), emotional states (e.g., anger, happiness, and sadness), or a combination of both. In most cases, the physiological and emotional changes happen naturally and not intended to circumvent the system. Within-speaker variability is considered as one of the main challenges that must be addressed by speaker identification systems that are prevalent in social robot applications such as a robot companion, a robot caregiver, or a robot personal assistant. Additionally, a person may intentionally alter his/her voice, an important factor contributing to within-speaker variability, to elude the system [8]. In addition, the performance of a speaker recognition system is affected by the technology used to capture, transmit, and save the speech signal. These scenarios are referred to as the channel variation effects, and the environmental, or background distortion. A significant research effort has been devoted to address this multifarious source of variations [9]. However, most of the research efforts focus on addressing the external-based sources of variation; considerable progress has been achieved in addressing the background noise (by using additive noise known as signal-to-noise ratio) and the channel variations (by using channel compensation techniques) [10]. Most of the feature extraction methods used in speaker recognition systems rely on spectral characteristic of the speech signal which is highly affected by a person’s health, age, and emotional state; the intrinsic-based source of variations. As such, this poses a huge challenge to speaker recognition systems particularly those intended for social robots. Among the ideal features of a speaker recognition systems are low discriminant power within-speaker variability, high discriminant power between-speaker variability, robust against the aforementioned source of variations (i.e., intrinsic-, external-, and conversation-based source of variations), easy to extract, and difficult to impersonate [3]. A significant research effort has been devoted to develop feature vectors that possess some of the above characteristics [11]. However, to the best of the authors knowledge no particular feature extraction method is robust against all sources of variations and all types of noises. In a parallel development, a large body of research has aimed at developing different speaker modeling, normalization, and adaption techniques that employ these feature vectors in order to increase the robustness of the speaker recognition system against the aforementioned source of variations [3].

Motivated by the above challenges, particularly those related to social robot applications, and noting that there is no single speaker recognition model that is universally applicable in different types of noise, different signal-to-noise ratios, and variable utterance lengths scenarios, and the absence of a “crystal ball” for speaker modeling, normalization, and adaptation, we propose a sophisticated architecture of speaker identification system for social robots. The proposed architecture encapsulates heterogeneous classifiers that employ prosodic and short-term spectral features in a two-stage classifier in order to integrate the advantages of using complementary features and different classifier models (generative and discriminative) in developing a robust speaker recognition system for social robots. The architecture utilizes gender information to cluster the population in the dataset into two groups (male and female) as well as deriving a speaker model from the gender-dependent model to provide strong coupling and improve recognition rate of the base classifier. Additionally, the system exploits the knowledge based-system (IF-THEN fuzzy rules), which is derived from the performance of the trained base classifier on limited size development set, to overcome the problem of limited size speech dataset which contains short utterances distorted with different types of noise.

The rest of the paper is organized as follows: in Section 2, we review the relevant literature and underscore the current state-of-the-art in speaker recognition systems. We also highlight the main challenges in speaker recognition. We present the detailed architecture of the proposed system in Section 3. The proposed methodology is evaluated via simulation studies in Section 4. The paper is concluded with a summary of the findings and the main characteristics of the present method in Section 5.

## 2. Related Works

Humans have the ability to recognize another person’s voice seamlessly without conscious effort. It is understood that various aspects of a person’s voice characteristics are implicitly and explicitly involved in the recognition process including spectral characteristics, prosody (syllable stress, intonation patterns, speaking rate and rhythm), and conversation-level features (lexicon and language). Analogous to humans, automatic speaker recognition systems employ various voice proprieties to recognize a person from his/her voice. These can be categorized into (1) short-term spectral features; (2) voice-based and prosodic features; (3) high-level features. The short-term spectral features are computed based on short frames in the range of 10–20 ms and can be seen as descriptors of vocal tract characteristics. Since this category of features require a small amount of data to be extracted, they fit well with real-time applications as in the case of speaker identification in social settings [11]. In social human–robot interactions, fast response which is prone to some degree of error is considered better than accurate but delayed response (i.e., humans prefer fast response with some level of error better than accurate but delayed response) [12]. Additionally, short-term spectral features are easy to extract and are text and language independent. Most of the automatic speaker recognition systems that have been developed in the last two decades employ short-term spectral features including mel-frequency cepstral coefficients (MFCC), linear predictive cepstral coefficients (LPCCs), line spectral frequencies (LSFs), perceptual linear prediction (PLP) coefficients, and gammatone frequency cepstral coefficients (GFCC), to name a few [11]. However, none of these features are robust to the above multifarious source of variations and background noise but may cooperate and complement each other [13,14]. A new feature extraction method has been proposed to construct features that have the advantages of cochleagram robustness and mel-spectrogram voiceprint richness [15]. mel-spectrogram and cochleagram are combined in two ways to construct two features, named MC-spectrogram and MC-cube. MC-spectrogram is constructed by superimposed mel-spectrogram and cochleagram on each other while MC-cube is constructed by mapping mel-spectrogram and cochleagram to a cube as harmonic mean. Several research studies suggest extension of MFCC features, which is based on mel-spectrogram, for speaker recognition system. The recognition rate has been improved by 4% by including delta derivatives of MFCC namely delta MFCC and delta-delta [16]. The best performance achieved was 94% recognition rate with MFCC feature vector having 18 coefficients. In [17], the 18 MFCC feature vector has been modified by adding two features. Observing and tracking spectral maxima in the energy spectrum of the speech signal were the key factor in calculating the two additional features. Adopting the modified MFCC feature vector has improved the recognition accuracy by 2.43% approximately. A huge research effort has been devoted to developing speaker modeling, including Gaussian mixture model (GMM) [18], GMM supervector with support vector machine (SVM) [19], and i-vector system [20,21], deep learning systems [6,22,23], normalization [24,25] and channel compensation techniques [26], and adaptation techniques [27,28] to reduce the effect of these variations on the performance of the speaker recognition system. Impressive progress has been achieved in addressing external-based source of variations, particularly channel variations and environmental and background distortion [9]. However, the performance of the state-of-the-art speaker recognition systems dramatically deteriorates when short utterances are used for training/testing particularly with low SNR [29,30]. In spite of the fact that deep neural networks (DNNs) provide a state-of-the-art tool for acoustic modeling, DNNs are data sensitive, and limited speech data as well as data-mismatch problems can deteriorate their performance [4,5,31].

In the last five years, deep learning methods have been demonstrated to outperform most of the classical speech and speaker recognition systems such as GMM-Universal Background Model (UBM), SVM, and *i*-vector [32,33]. However, deep learning systems require huge speech databases to be labeled and trained; theses databases also need to include phonetically rich sentences or at least phonetically balanced sentences [31]. In addition, most of speaker recognition systems that were developed based on deep learning techniques have been applied to text-dependent speaker verification tasks [4]. Hence, training deep learning systems on limited data is a difficult task and may not necessarily lead to speaker recognition systems with state-of-the-art performance. The above-mentioned challenges which are frequently encountered in some of the social robot applications such as social robots for people with developmental disabilities, elderly care housing and assisted living, nursing robots [34,35,36] hinder the direct application of deep learning systems. In such scenarios, collecting large databases is time-consuming, strenuous, inconvenient, expensive, and may be an impossible task as in the case of people with speech impairments. The key advantage of deep learning systems that they can recognize a large number of subjects may not justify their adaptation for speaker recognition for social robots as social robots need to interact with a limited number of persons.

Some research studies suggest that the performance of GMM-UBM system is close to *i*-vector based system in short duration utterance and over perform it in very short utterances (less than 2 s) [37]. Another challenge that needs more work to address is intrinsic-based source of variations [9] and synthesis and conversion spoofing attacks [38]; voice conversion and statistical parametric speech synthesizers that may use spectral-based representation similar to the one used in speaker recognition systems that employ spectral features.

The high-level features use a speaker’s lexicon (i.e., the kind of words that a speaker frequently uses in his/her conversations) to extract a signature that characterizes a speaker. Some research studies show that this category of features is robust against channel variation and background noise, but it requires substantial computational cost and is difficult to extract [3,39]. Additionally, this category is language and text dependent, needs a lot of training data, and is easier to impersonate. The pros and cons of the prosodic features category sit in the middle of the scale between high-level feature and short-term spectral feature. Researchers suggest that the prosodic features carry less discriminant power than short-term spectral features but are complementary [3]. However, due to the nature of prosody, which reflects the differences in speaking styles such as rate, rhythm, and intonation pattern. This category shows more resistance to voice synthesis and conversion spoofing attacks, but it is valuable to human impersonation. Employing this feature in speaker recognition systems provides robustness to adversarial attacks to which most deep speaker recognition systems are prone to [40]. One can argue that a speaker recognition system that employs short-term spectral and prosodic features is more robust than those systems that employ only one type of these features. A reasonable research effort has been devoted to fuse prosodic and spectral features in order to improve the accuracy and robustness of the recognition of a speaker age, gender, and identity [41,42,43]. However, most of these systems adopt either fusion at score level for prosodic-based system and spectral-based system or fusion at feature level by stacking prosodic-based feature representation with spectral-based feature representation. Fusion at score and feature level has been demonstrated in [44]; the fusion at score level was presented as a fusion of the outputs of two prosodic-based classifiers and the output of one cepstral-based classifier while fusion at feature level was performed by stacking cepstral *i*-vector with a combination of the two prosodic *i*-vector representation. Some prosodic features, particularly pitch frequency F0, have demonstrated excellent performance in gender classification tasks [45]. Gender information can be used to enhance the GMM-based speaker recognition system in two ways. First, adaptation of speaker-dependent GMM from gender-dependent GMM-UBM is computationally efficient and demonstrates stronger coupling than adaptation of speaker-dependent GMM from gender-independent GMM-UBM [27]. Second, Reynolds et al. [46] demonstrated that increasing the population size degrades the recognition accuracy of GMM-based speaker identification systems. Therefore, exploiting gender information to cluster speaker population into two groups reduces the population size and consequently improves the recognition accuracy of GMM-based speaker identification systems [47]. Constructing cluster-based GMM for speaker populations improves the performance of speaker recognition systems as demonstrated in [48]. A combination of prosodic features are exploited to cluster speaker populations into male and female groups to enhance the performance of emotional speech classification systems [49]. Adopting a gender-dependent parameterizations approach to construct a GMM-based speaker recognition system improves the performance of the system, namely equal error rate and half total error rate.

The contribution of this study within this context is presentation of a novel architecture of a speaker identification system for social robots that employs prosodic features as well as two types of spectral-based features (MFCC and GFCC) in order to enhance the overall recognition accuracy of the system. The system works in two stages; in the first stage, a binary classifier exploits the superiority of prosodic features to infer gender information and reduce the size of gallery set by clustering the speaker population into two groups. In the second stage, the outputs of four classifiers are fused in a novel way to improve the overall performance of the system; particularly in the case of short duration utterances and low SNR which is a common condition in speaker identification in social settings.

## 3. Overview of the Proposed Architecture

**Figure 1:**
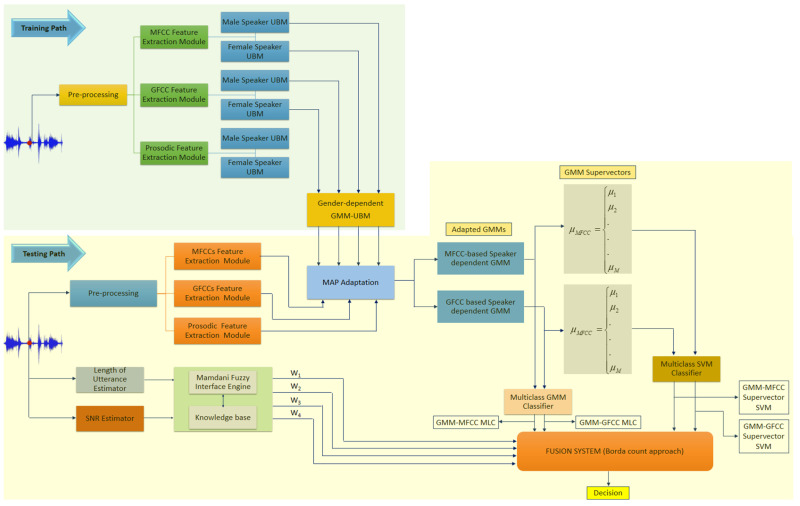
The architecture of the proposed speaker recognition system.

The proposed architecture of the speaker recognition system consists of two classifiers working in a quasi-parallel fashion. The overall architecture of the system is depicted in Figure 1. The upper section represents the enrollment process (training path), whereas the lower part elaborates the recognition process (testing path). The function of the feature extraction module is to transform the speaker’s utterances into feature vectors that contain his/her specific characteristics. As shown in Figure 1, the feature extraction module is common to both enrolment process (training) and identification process (testing). In the enrolment process, the speaker’s model is trained by the feature vectors that were extracted from speech utterances by a target speaker and labeled accordingly. The recognition process is performed by extracting feature vectors from an unknown speaker’s utterance which in turn is used to build the unknown speaker model. This model is subsequently matched to one of the labeled speaker models that were constructed during the enrolment process. One may infer that the feature extraction processes for both classifiers are initiated in parallel and at the same time. However, the second classifier requires gender information as well as MFCC and GFCC feature vectors in order to complete the identification process. The first classifier is a binary SVM classifier that uses prosodic feature to determine the gender of the speaker. The second classifier, which is a combination of GMM-based classifier and GMM supervector-based SVM classifiers, employs MFCCs and GFCCs feature as well as gender information to determine the identity of the speaker. In order to compute the GMM supervectors for both types of feature vectors (i.e., MFCCs and GFCCs supervectors), speaker’s gender must be known. As shown in Figure 1, a binary SVM relies upon prosodic features to determine the gender of the speaker who utters a speech.

The proposed speaker identification system works in two stages: first, the prosodic feature vector is used to determine if an utterance originated from a male or a female speaker. A binary SVM is trained using a prosodic feature vector to classify the utterance into two classes (males and females). The proposed architecture incorporates the outcome of the first stage (gender classification) into the second stage where MFCCs and GFCCs feature vectors that are extracted from the same utterance are used to derive speaker-dependent GMM from a pre-trained gender-dependent GMM-UBM. The gender-dependent GMM-UBM is trained by utterances originating from specific gender groups of speakers (male or female group). The speaker-dependent GMM derived from gender-dependent GMM-UBM shows excellent coupling to the gender-dependent GMM-UBM and requires low computational power and less time as compared with the model derived from gender-independent GMM-UBM (i.e., the GMM-UBM is trained by utterances from male and female speakers). The resultant speaker-dependent GMMs are used to create GMM-supervectors by stacking the mean vectors of the speaker-dependent GMMs. As shown in Figure 1, four classifiers were developed by employing GMMs and GMM-supervectors. Two of these classifiers are generative-based classifier. The first one is a maximum likelihood classifier (MLC) that employs GMMs trained by MFCC feature vectors and the second classifier is an MLC that employs GMMs trained by GFCC feature vectors. The third and the fourth classifiers are discriminative-based classifiers; namely, SVM that employs GMM-supervectors derived from GMM trained by MFCC and GFCC feature vectors, respectively.

Fusion at score level was adopted to combine the outputs of all the aforementioned classifiers into a single score, namely the weighted Borda count method (please see: https://en.wikipedia.org/wiki/Borda_count, accessed on 12 July 2021). In this study, the weighted Borda count method is a plausible choice as it does not require transforming all the scores of the base classifier into a common domain (i.e., no normalization process). Additionally, the fusion system exploits the classes ranking information of the base classifiers on the development set to compute the weights for the base classifiers (more details in Section 3.3). Borda count method uses the match scores of the base classifiers to arrange the classes in descending order. Then, for each class, the Borda count is represented as the sum of the number of classes ranked below it by the respective classifier. The higher the magnitude of Borda count for a class, the greater the degree of agreement by the base classifiers that the test sample belongs to that class [49]. We propose a novel way to account for the Borda count for each classifier by a Mamdani fuzzy inference system [50]. Since we know that certain classifiers are more likely to outperform others at specific conditions, the weight factors are computed via Mamdani fuzzy inference system to exploit the individual classifier capabilities at those conditions.

The fuzzy inference system employs the knowledge about the recognition rate trend of the aforementioned classifiers when they are evaluated on development set. This information is used to derive a set of rules in the form of IF-THEN fuzzy rules based on Mamdani fuzzy inference engine in order to compute the weighting factors for all the aforementioned classifiers such that the overall recognition rate is improved. Here, we employed the recognition rate of each classifier as a function of length of utterance and the signal-to-noise ratio (SNR). Then, for each combination of the length of utterance and the SNR, a respective rule is derived taking into consideration that each classifier is weighted by a factor proportional to its recognition rate. Additionally, the fuzzy rules consider the selected classifiers to be combined to complement each other from the perspectives of feature types and classifier model with priority to feature type.

In the testing path, the feature extraction modules of all feature vectors used by the two classifiers (i.e., speaker’s gender and identity classification) are initiated at the same time. Thus, there is no noticeable delay caused by this architecture (i.e., the second classifier needs the output of the first classifier (i.e., male or female) as one of its inputs in order to identify a speaker).

### 3.1. Feature Extraction Modules

**Figure 2:**
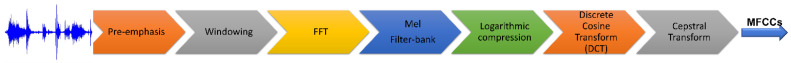
Block diagram of MFCCs feature extraction modules.

**Figure 3:**
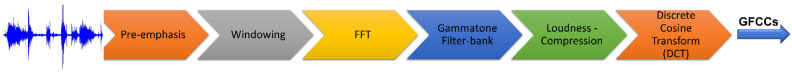
Block diagram of GFCCs feature extraction module.

The proposed system exploits two groups of voice-based features to identify a speaker, namely prosodic features and spectral features. The GFCC and MFCC features are adopted as spectral features. The modular representation of GFCCs and MFCCs feature extraction methods are depicted in Figure 2 and Figure 3, respectively. As shown in these figures, both MFCCs and GFCCs share the same stages except the type of the filter-banks that are applied to the resultant frequency domain signal from fast Fourier transform (FFT) and the compression operation. The MFCCs feature extraction method applies mel filter-bank after FFT stage and followed by logarithmic compression and discrete cosine transform (Section 3.1.1). On the other hand, in the GFCCs feature extraction method, the gammatone filter-bank is applied to the resultant frequency domain signal from FFT before loudness compression and discrete cosine transform take place (Section 3.1.2). The prosodic feature vector characterizes four areas of prosody including pitch, loudness, voice quality, and formant. Pitch and loudness information were represented as statistical measure of fundamental frequency (F0) and energy, respectively. Harmonics-to-noise ratio (HNR), jitter, and shimmer represent voice quality measurements while the first three formants characterize the fourth category of prosodic information. The complete details about prosodic feature vector are discussed in Section 3.1.3.

#### 3.1.1. MFCCs Feature Extraction Module

**Figure 4:**
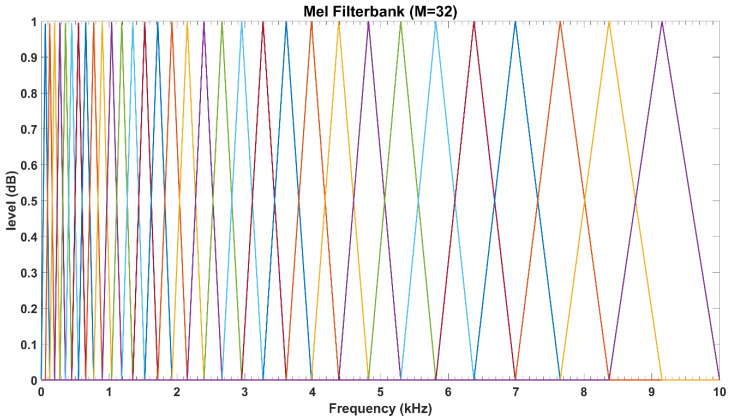
The triangular mel-frequency scaled filter-banks.

MFCCs feature vector is determined in accordance with the notion that the frequency range of human spoken words is generally limited to 1000 Hz. Therefore, MFCCs employ linearly spaced filters at below this threshold frequency and logarithmic spaced filters at above this frequency. This implies that the filter-bank selected the most appropriate frequency range of interest, i.e., more filters in the narrow bandwidths below 1000 Hz and fewer number of filters at outside this range as shown in Figure 4.

Figure 2 demonstrates that the first step in the extraction process is to boost the input speech signal as:

(1) Y(n)=X(n)−a×X(n−1)

where X(n) is the input speech signal, Y(n) is the boosted speech signal. The interval of the pre-emphasized factor is selected within the range [0.95, 0.98]. Subsequently, we apply a smooth window function (hamming windows) to the pre-emphasized speech signal Y(n):

(2) $$\;\;\;\;\;W\left(n\right)=0.54-0.46\times \cos\left(\frac{2\pi n}{N-1}\right),\;0\leq n<N-1$$

**Figure 5:**
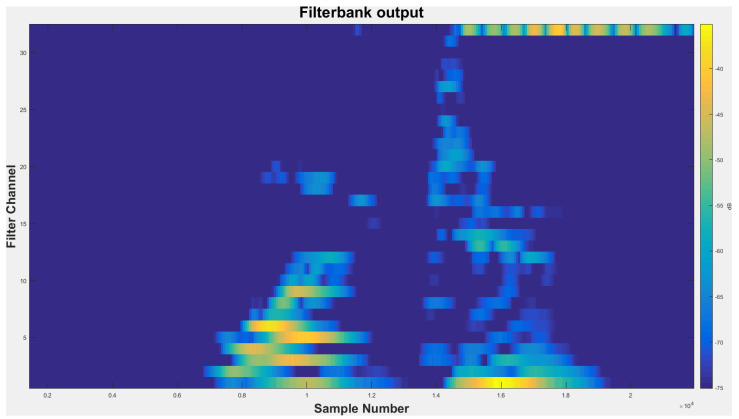
The output of mel-frequency filter-banks.

We then transform the resulting time-domain signal to frequency domain via the ubiquitous fast Fourier transform (FFT). As expected, the FFT process generates a fluctuating spectrum. Thus, in order to ensure that the FFT spectrum extracts the most relevant information for speaker recognition, we design the filter-bank based on the mel scale. Figure 5 shows the output of the mel-frequency filter-banks. Here, we apply logarithmic compression and discrete cosine transform as in (3) to obtain the MFCCs. The main objective of the discrete cosine transform is to transform log mel spectrum into time domain.

(3) $$C_{n}=\overset{M}{\underset{m}{\sum }}\left[\log S\left(m\right)\right]\cos\left[\frac{\pi n}{M}\left(m-\frac{1}{2}\right)\right]$$

where S(m), m=1,2….,M is the output of an M-channel filter-bank, n is the index of the cepstral coefficient. For the purpose of this project, we retained the 12 lowest $C_{n}$ excluding the 0th coefficient.

#### 3.1.2. GFCCs Feature Extraction Module

The GFCCs feature vector is computed in the same way as MFCCs feature vector as shown in Figure 3. The key difference between the GFCCs and MFCCs is that the GFCCs feature extraction uses a bio-inspired gammatone filter-bank to extract the most discriminant information from FFT spectrum, which was originally designed to model the human auditory spectral response, particularly modeling the auditory processing at the cochlea. Like MFCCs, the speech signal is pre-emphasized first and followed by Windowing and FFT stages. Then, the gammatone filter-bank is applied to the resultant FFT spectrum. The impulse response of each filter in gammatone filter-bank can be represented as in (4) [51].

(4) $$g\left(t\right)=at^{n-1}e^{-2\pi bt}\cos\left(2\pi f_{c}t+\varphi \right)$$

Since a is constant, n and φ are fixed for the entire filter-bank. The frequency selectivity of gammatone filter-bank is mainly defined by central frequency, f, and the filter’s bandwidth b. A suggested method to compute the central frequency and the filter’s bandwidth is by using an approximation to the bandwidth of human auditory filter at the cochlea. Equivalent rectangular bandwidth (ERB), which represents the bandwidth of series rectangular filters that are used to model the human cochlea, can be used to compute the filter’s bandwidth and its central frequencies. Moore [52] modeled the human auditory system using ERB as:

(5) $$\mathrm{ERB}\left(f_{c}\right)=24.7+0.108\;f_{c}$$

The idea of ERB is adopted by Patterson et al. [53], to estimate the bandwidth and the center frequencies of the gammatone filter. It has been suggested that the two parameters of gammatone filter (the bandwidth, b,  and order of the filter, n) should be set as b=1.019 ERB and n=4 in order to attain a filter with good match to human auditory filter.

As suggested by Moore [52], the center frequencies of the gammatone filter are equally spaced on the ERB frequency scale. The relationship between the number of ERBs to the center frequencies, $f_{c}$, can be expressed as:

(6) $$number\;of\;ERBs=21.4\;log_{10}\left(0.00437\;f_{c}+1\right)$$

**Figure 6:**
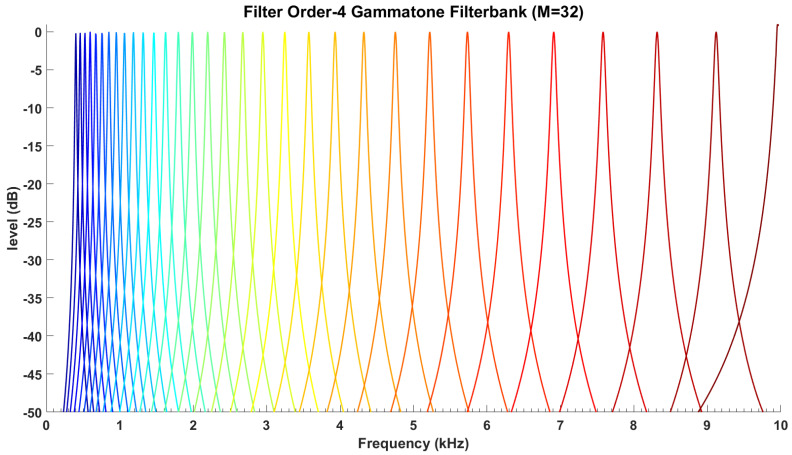
The frequency response of gammatone filter-banks.

**Figure 7:**
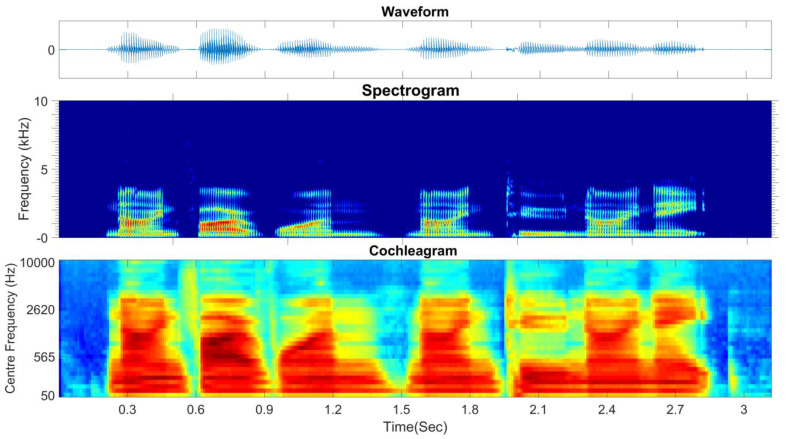
The spectrogram and cochleagram of a sample speech signal from TIDIGITS database.

The ERB scale, which is approximately logarithmic, is defined as the number of ERBs below each frequency. This scale correlates the center frequencies with 1f distribution of frequency energy of speech signal. In other words, the frequency-dependent bandwidth of gammatone filter produces a narrower filter at low frequencies and a broader filter at high frequencies as shown in Figure 6 (fourth-order gammatone filter-bank with 32-channel outputs). In this study, the fourth-order gammatone filter-bank with 64-channel outputs is used to extract the GFCCs feature vectors. The GFCCs feature vectors are obtained by applying a cubic root operation (loudness-compression) and then de-correlating the feature components by applying a discrete cosine transform. Additionally, we retained the 22 lowest coefficients excluding the 0th coefficient. The gammatone filter-bank response (termed the cochleagram) and typical spectrogram in response to a sample of speech signal from TIDIGITS are depicted in Figure 7.

#### 3.1.3. The Prosodic Feature Extraction Module

It has been documented that 90% of speaker recognition systems have employed short-term spectral features such as MFCC, LPCC, and GFCC which have been found to carry high power discriminant information for the speaker recognition task [11]. While the short-term spectral features span short frames of about 10–30 ms and contain information correlating to timber and resonance proprieties of vocal tract, the prosodic features span longer frames of about 10 to hundreds of milliseconds and correlate with non-segmental aspect of speech such as intonation, stress, rate and rhythmic organization of the speech. Since prosodic features span over long segments like syllables, words, and utterances, it is believed to contain complementary information and have more robustness for channel and background distortion. Different combinations of the prosodic parameters have been used widely for language and emotion identification, age and gender classification, and speaker authentication. The fundamental frequency (F0) is the most important parameter among these prosodic parameters. Here, selection of prosodic features from previous works of [42,54,55] was adopted as feature vectors for first-stage classifier (gender classification). Particularly, various statistical measures of fundamental frequency (F0), spectral centroid (SC), spectral flatness measure (SFM), Shannon entropy (SE), harmonics-to-noise ratio (HNR), jitter and shimmer, and the first three formants were adopted to construct one prosodic feature vector. Individually, these features are correlated with pitch accents and boundary tones, the approximate location of formants, the flatness of the spectrum, the degree of randomness of spectral probability density, the amount of noise in the speech signal, the overall periodicity of speech signal, variability of fundamental frequency, voice pathologies, and psychological characteristics of vocal tract (length, shape, and volume), respectively. HNR and the first three formant frequencies are calculated with the VoiceSauce feature extraction tool [55]. SC, SFM, and SE are computed with MIR toolbox [56]. Absolute jitter and shimmer measurements were extracted by using Praat voice analysis software [57]. The complete list of features and the corresponding statistical measures that were applied to construct the prosodic feature vector are listed in Table A1 (Appendix A).

### 3.2. Speaker Modeling (Classification Algorithms)

In this section, for the sake of completeness of the paper, we consider popular classification algorithms that are widely used in speech recognition and speaker identification.

#### 3.2.1. Gaussian Mixture Model

The well-known GMM approach was adopted to construct the first two classifiers; namely GMM-MFCC MLC and GMM-GFCC MLC. We extracted the feature vectors for the MFCC and GFCC from the speech data of all speakers in training data (gallery set). For each of these feature vectors, two speaker-independent world models (a well-known UBM) were created; the first UBM is trained by the feature vector extracted from female speakers and the second UBM is trained by the feature vector that is extracted from male speakers. The UBM is estimated by training M-component GMM with the standard expectation–maximization (EM) algorithm [28]. The UBM represents speaker-independent distribution of the feature vectors. In this study, we employ 256-compnenent GMM to build the UBM. The UBM is represented by a GMM with 256-compnents, denoted by $\lambda_{UBM}$, that is characterized by its probability density function as:

(7) $$p\left(\vec{x}|\lambda \right)=\overset{M}{\underset{i=1}{\sum }}w_{i}p_{i}\left(\vec{x}\right)\;$$

The model is estimated by a weighted linear combination of D-variate Gaussian density function $p_{i}\left(\vec{x}\right)$, each parameterized by a mean D×1 vector, $\mu_{i}$, mixing weights, which are constrained by $w_{i}\geq 0$, $\overset{M}{\underset{i=1}{\sum }}w_{i}=1$, and a D×D covariance matrix, $\Sigma_{i}$ as:

(8) $$p_{i}\left(\vec{x}\right)=\frac{1}{2\pi^{D/2}{\left|\Sigma_{i}\right|}^{1/2}}exp\{\frac{1}{2}{\left(x-\mu_{i}\right)}^{\prime }(\Sigma_{i}{}^{-1})\;\left(x-\mu_{i}\right)\}\;$$

The training of UBM is to estimate the parameters of 256-component GMM, $\lambda_{UBM}={\{w_{i},\mu_{i},\Sigma_{i}\}}_{i=1}^{M}$, from the training samples. Subsequently, for each speaker in the gallery set, we apply maximum a posteriori (MAP) to estimate the specific GMM from UBM-GMM. Since the UBM represents speaker-independent distribution of the feature vectors, the adaptation approach facilitates the fast-scoring as there is a strong coupling between speaker’s model and the UBM. Here, the gender-dependent UBMs were constructed to provide stronger coupling and faster scoring than that of gender-independent UBM. It should also be noted that all or some of GMM’s parameters ($\lambda_{UBM}=\{w,\mu ,\Sigma \}$ can be adapted by a Maximum A Posteriori (MAP) approach. Here, we adapted only the mean μ to represent specific speaker’s model. Now, let us assume a group of speakers s=1,2,3,…, S represented by GMMs $\lambda_{s}=\lambda_{1},\lambda_{2},\lambda_{3},\ldots ,\lambda_{S}$. The goal is to find the speaker identity $\hat{s}$ whose model has the maximum a posteriori probability for a given observation $X_{k}=\{x_{1},\ldots ,x_{T}\}$ (MFCC or GFCC feature vector). We calculate the posteriori probability of all observations $X_{k}=X_{1},X_{2},X_{3},\ldots ,X_{K}$ in probe set against all speaker models $\lambda_{s}=\lambda_{1},\lambda_{2},\lambda_{3},\ldots ,\lambda_{S}$ in gallery set as (9). As sandk vary from 1 to number of speakers in gallery set and number of utterances in probe set, respectively, the result from (9) is S×K matrix.

(9) $${P_{r}(\lambda_{s}|X_{k})=\frac{p(X_{k}|\lambda_{s})}{p\left(X_{k}\right)}P_{r}\left(\lambda_{s}\right)|}_{\begin{array}{c} 1\leq s\leq S\\ 1\leq k\leq K \end{array}}{}_{}\;$$

Assuming equal prior probabilities of a speaker, the terms $P_{r}\left(\lambda_{s}\right)$ and $p\left(X_{k}\right)$ are constant for all speaker, thus both terms can be ignored in (9). Since each subject in the probe set is represented as $X_{k}=\{x_{1},\ldots ,x_{T}\}$, thus by using logarithmic and assuming independence between observations, calculation of posteriori probability $P_{r}$ can be simplified as (10). The outputs of the two GMM-based classifiers (GMM-MFCC MLC and GMM-GFCC MLC) were computed using (10).

(10) $$P_{r}(\lambda_{s}|X_{k})=\overset{T}{\underset{t=1}{\sum }}log\;{p(x_{k}^{t}|\lambda_{s})|}_{\begin{array}{c} 1\leq s\leq S\\ 1\leq k\leq K \end{array}}{}_{}\;$$

#### 3.2.2. GMM Supervector and Support Vector Machine

One of the challenges of exploiting information in voice modality is that the utterances are manifested with varying time durations. The dimension of feature vectors depends on the time duration of these utterances; hence the resultant feature vectors from feature extraction modules (i.e., MFCC, GFCC, prosodic) have variable dimensions. Since most of the discriminant classifiers including SVM require fixed length feature vector as input, the speaker recognition research community has discovered a way to represent these time-varying utterances as a fixed-length feature vectors. The method relies upon using the parameters of speaker-dependent GMM. A speaker can be modeled as M-component GMM either by adapting a specific speaker model from UBM-GMM using MAP or by training M-component GMM with EM algorithm independently from UBM-GMM. Deriving a speaker-dependent model by adaptation approach provides a good coupling between a speaker model and UBM-GMM. Since the UBM-GMM represents a distribution of all speakers in the galley set, this coupling is desirable. Additionally, the adaptation approach reduces the computational cost of building a speaker-dependent model and facilitates real-time response.

In speaker recognition literature, supervector refers to a fixed length feature vector constructed by combining many smaller-dimensional vectors into a higher-dimensional vector. In this study, GMM supervector is constructed by concatenating d-dimensional mean vector of the M-component speaker-dependent model that is adapted from pre-trained UBM-GMM. The resultant GMM supervector with M∗d dimension is fed to SVM. The dimension of the MFCC-GMM supervector is 3072 (d=12 and M=256) and the dimension of the GFCC-GMM supervector is 5632 (d=22 and M=256). Principal component analysis is applied to reduce the dimension of thesesupervector before being fed to SVM. We refer to the two classifiers that were trained by MFCC-GMM supervector and GFCC-GMM supervector as MFCC-GMM supervector SVM and GFCC-GMM supervector SVM, respectively.

SVM is one of the most powerful discriminative classifiers with excellent generalization performance to classify any unseen data. Basically, SVM is a supervised binary classifier that aims to separate the two classes by modeling a decision boundary as hyperplane; hence, adopting SVM to solve speaker verification is sensible. In speaker verification, the task is to determine if a given utterance matches or does not match a target model (claimant identity). In the training stage, all training feature vectors that are extracted from the target speaker’s voice samples are represented as one class and the second class is represented by all training feature vectors that are extracted from the background “impostor” speaker’s voice samples. SVM maps the training vector to high-dimensional space and finds an optimum hyperplane that separates the two classes (i.e., target speaker and impostor) with maximum margin. Since speaker identification is a multiclass classification problem, the well-known method One-Vs-All (OVA) SVM is adopted to extend the binary SVM to accommodate the multiclass classification task. Adopting the OVA approach, which requires constructing as many binary SVM classifier as the number of classes, fits our framework of integrating the outputs of various classifiers. The output of SVM should be represented as score vector that can be interpreted either as the degree of match between a given utterance and every speaker’s voice signature in gallery set or as the probability that a given utterance originates from every speaker in the gallery set. The outputs of multiclass SVM that are constructed by the OVA approach can be expressed as probabilistic outputs; hence, the OVA is adopted to construct multiclass SVM classifier. The probabilistic outputs are used to rank the classes and compute the Borda count value for each class.

In the proposed speaker recognition system, there is no need to unify and transform the outputs of the base classifiers to common domain. However, the outputs of the base classifiers should be expressed as scores (represent the degree of support) that are used to rank all the classes in descending order. The outputs of SVM, which are mostly expressed as a label for the predicted class that a test sample is assigned to (for example, the output of binary classifier is either +1 or −1), are not compatible with our fusion system. Therefore, the method suggested by Platt [58] is used to estimate probabilistic outputs for SVM classifier. The discriminative function of binary SVM can be expressed as (11) [59]:

(11) $$f\left(x\right)=\overset{N}{\underset{i=1}{\sum }}\alpha_{i}y_{i}k\left(x,x_{i}\right)+d\;$$

where $y_{i}$ is either +1 or−1, and represents ideal output, $x_{i}$ is support vector, d is bias, $k\left(x,x_{i}\right)$ is kernel function, α is weight, $\overset{N}{\underset{i=1}{\sum }}\alpha_{i}y_{i}=0\;\mathrm{and}\;\alpha_{i}>0$. The kernel function satisfies the Merce condition, so that k(x,y) can be expressed as (12):

(12) $$k\left(x,y\right)=\Phi {\left(x\right)}^{T}\Phi \left(y\right)\;$$

where Φ(x) is a mapping from input space (feature vector space) to high-dimensional space. Here, a radial basis function is selected as SVM kernel function and 3-fold cross validation was adopted to find best parameters for it. Mapping input feature vectors to high-dimensional space by using “kernel trick” which implicitly transforms the input vectors to high-dimensional space without explicit computation of dot product in high-dimensional space. Hence, all the dot products in SVM computations are replaced with kernel function k(x,y). This implies that SVM optimizes a decision boundary (hyperplane) between the two classes in high-dimensional space without explicit computation of Φ(x). For more information about adopting SVM in speaker recognition, the reader may refer to [60], and to [59,61] for more in-depth information about the SVM and kernel functions.

### 3.3. Fusion System

A large number of biometric authentication systems have adopted fusion of information at the score level to improve the overall performance of authentication systems. These systems employ various biometric modalities, different classification architectures, and feature extraction approaches. The key is not only to generate a set of feature vectors that complement each other but also to develop classifiers with satisfactory performance in diverse and challenging conditions. Here, the scores of the four classifiers (GMM-MFCC MLC, GMM-GFCC MLC, GMM-MFCC supervector SVM, and GMM-GFCC supervector SVM) are integrated using weighted Borda count method. It is worth noting that these classifiers represent both generative and discriminative approaches which intuitively can be seen as highlighting similarities and differences between classes, respectively. The weight factors are computed on the fly using a Fuzzy rule-based inference engine. The knowledge base of the fuzzy inference system is represented as IF-THEN fuzzy rules. These rules are derived by studying the recognition rate of the aforementioned classifiers as a function of SNR and the length of utterance.

**Figure 8:**
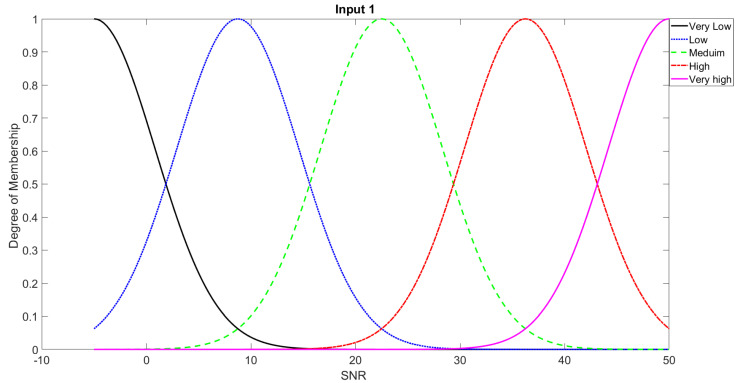
Fuzzy membership function for SNR input.

**Figure 9:**
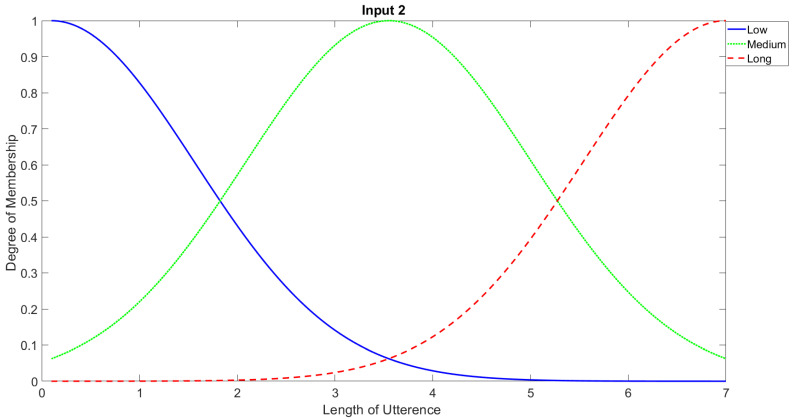
Fuzzy membership function for length of utterance input.

**Figure 10:**
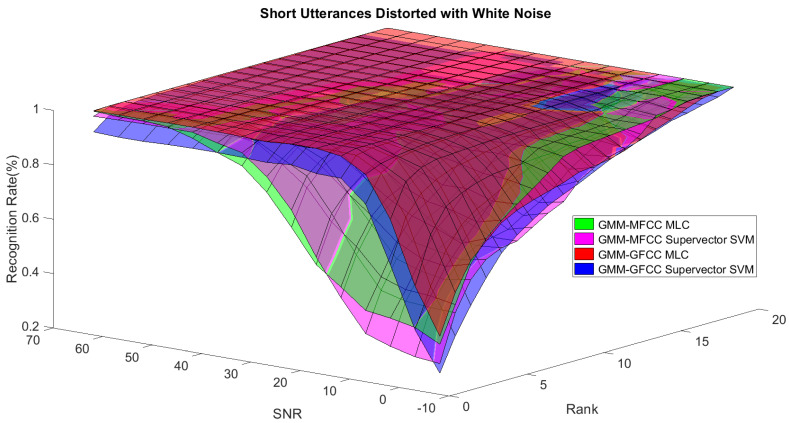
The 3D surface of the recognition rates for the four base classifiers on short utterances distorted with white noise.

**Figure 11:**
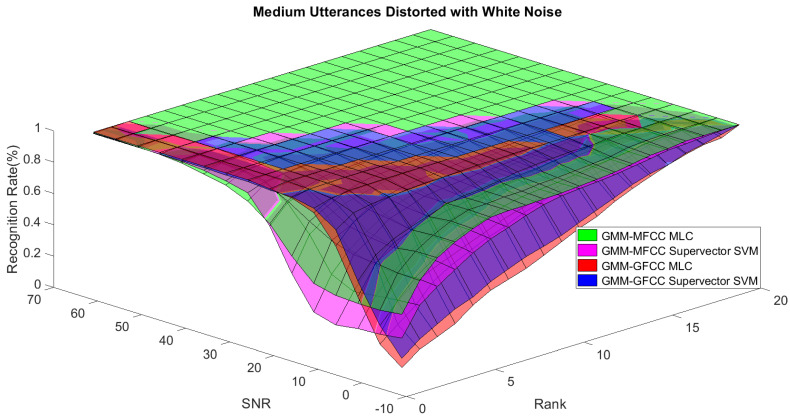
The 3D surface of the recognition rates for the base classifiers on medium utterances distorted with white noise.

**Figure 12:**
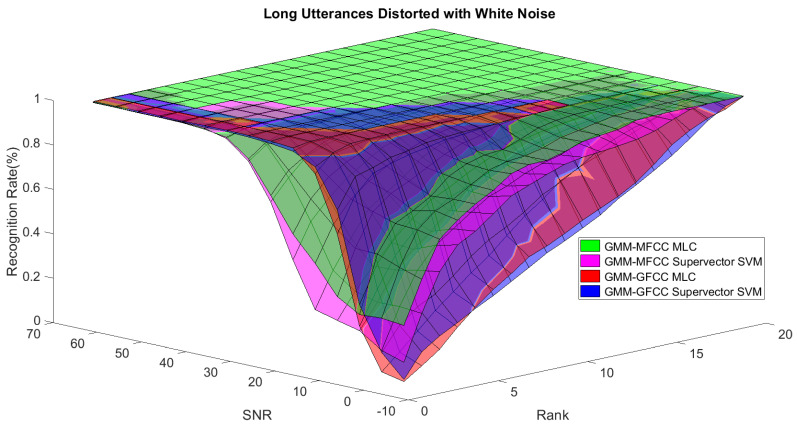
The 3D surface of the recognition rates for the base classifiers on long utterances distorted with white noise.

**Figure 13:**
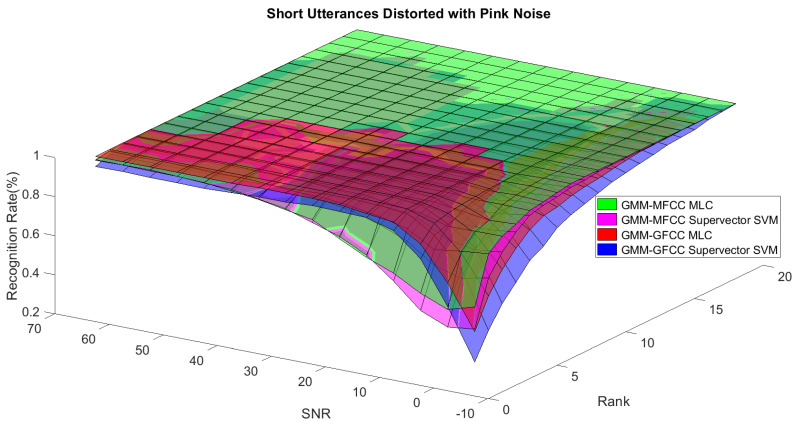
The 3D surface of the recognition rates for the base classifiers on short utterances distorted with pink noise.

**Figure 14:**
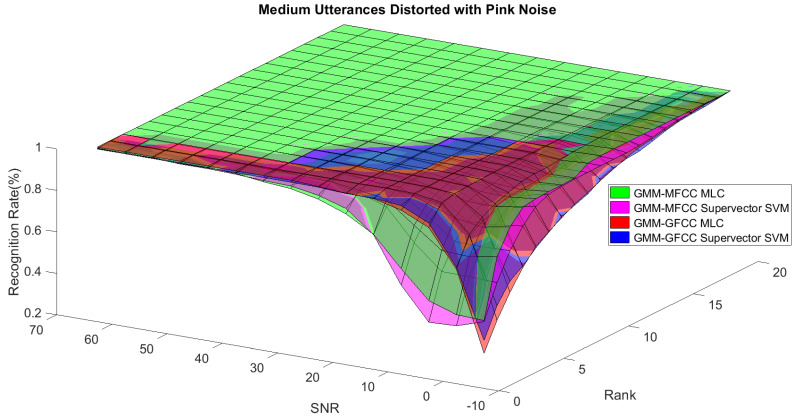
The 3D surface of the recognition rates for the base classifiers on medium utterances distorted with pink noise.

**Figure 15:**
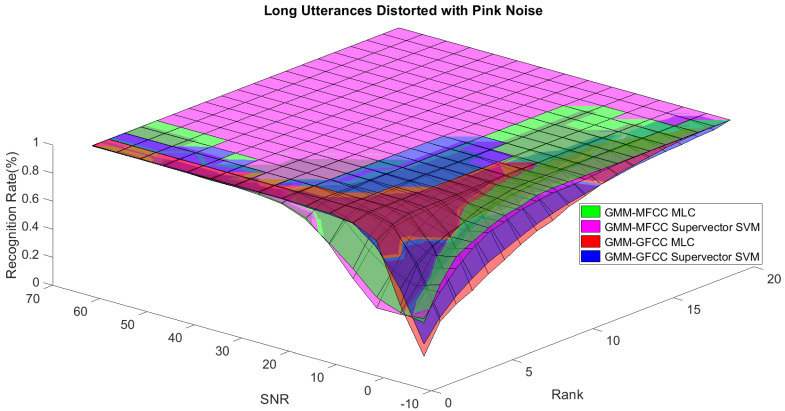
The 3D surface of the recognition rates for the base classifiers on long utterances distorted with pink noise.

**Figure 16:**
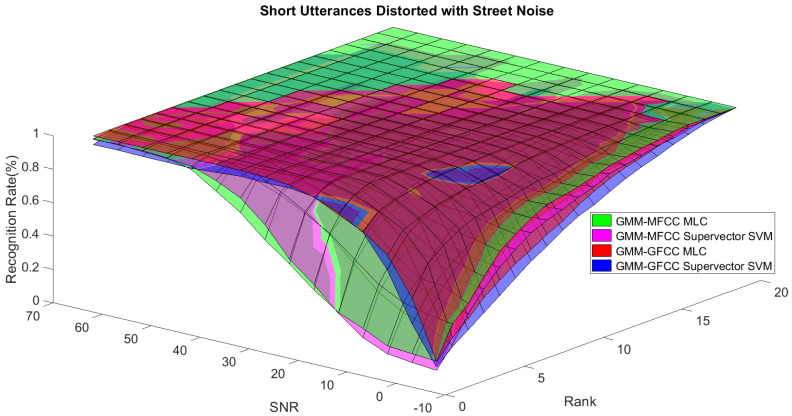
The 3D surface of the recognition rates for the base classifiers on short utterances distorted with street noise.

**Figure 17:**
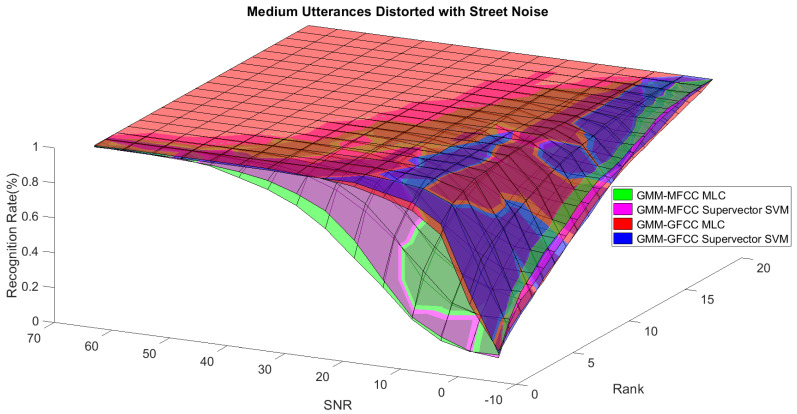
The 3D surface of the recognition rates for the base classifiers on medium utterances distorted with street noise.

**Figure 18:**
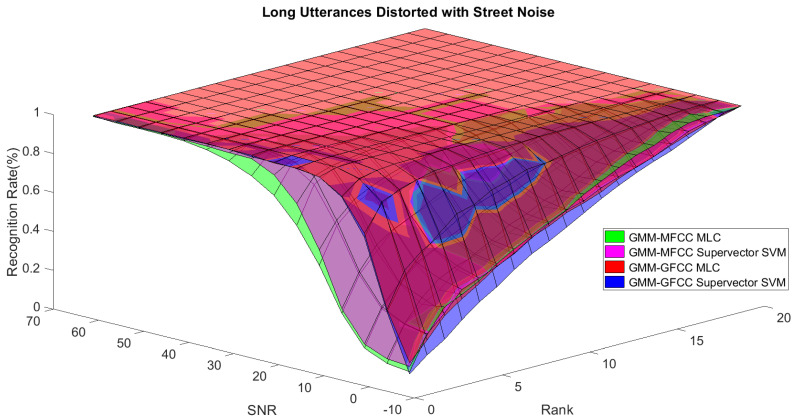
The 3D surface of the recognition rates for the base classifiers on long utterances distorted with street noise.

In order to study the recognition behavior for individual classifiers as a function of SNR and the length of utterance, the TIDIGITS corpus [62] was divided into three equal sets; training set, development set, and testing set. The training set was used to train individual classifiers independently. All utterances in development set and testing set were distorted by three types of noise: white Gaussian noise, pink and street noise with SNR range from −5 to 50 dB, in increments of 5 dB. Additionally, all utterances in the development set were categorized into three groups (short, medium, long) based on the length of utterance. Then, the recognition rates of these trained classifiers were computed on each group and depicted as a function of SNR and rank. Each group (i.e., short, medium, long) contains utterances with range of time duration. Additionally, the estimation of SNR is prone to error. Thus, we fuzzified SNR and length of utterance by designing membership functions for each of them empirically as depicted in Figure 8 and Figure 9, respectively. The SNR and length of utterance represent inputs of the fuzzy inference system and the weight factors represent the outputs of the fuzzy inference system as shown in Figure 1. The statistical measures of time duration of all utterances (i.e., min, max, mean) in each group were used to facilitate determining the parameters of membership function of length of utterance. The SNR membership function parameters were determined empirically based on the performance of the base classifiers on different noise levels. For each type of noise, a set of parameters was selected relying upon the performance of the base classifiers. The knowledge base, which is represented as IF-THEN fuzzy rules, is derived from the performance of the base classifiers on the development set that categorized into three groups and distorted with three types of noise (white, pink, street). The performance of base classifiers on a combination of the three types of noise with three categories of length of utterance (i.e., total of nine groups from the development set) are depicted in Figure 10, Figure 11, Figure 12, Figure 13, Figure 14, Figure 15, Figure 16, Figure 17 and Figure 18. For each type of noise, a set of IF-THEN rules are derived relying upon the performance of the base classifiers shown in Figure 10, Figure 11, Figure 12, Figure 13, Figure 14, Figure 15, Figure 16, Figure 17 and Figure 18. These figures depict the relationship between the recognition rate of the system and SNR at various ranks. Since each of the base classifiers return a ranked list of candidates based on their match scores, recognition rate of each classifier can be calculated at different rank. When recognition rate is calculated considering the top ranked candidate (i.e., the candidate with best score is the correct class), it is called rank-1 recognition rate. Additionally, when recognition rate is calculated considering the first and second top ranked candidates (i.e., the correct class is among the two top candidates), it called rank-2 recognition rate and so on. The Borda count method uses ranking information to determine the winner class, thus, change in recognition rates of the base classifiers with respect to rank (rank axis in Figure 10, Figure 11, Figure 12, Figure 13, Figure 14, Figure 15, Figure 16, Figure 17 and Figure 18) is exploited in deriving IF-THEN rules. The rules are derived such that more weight is given to the base classifier that demonstrates a big improvement in recognition rate with respect to rank axis. For example, by studying the performance of the base classifiers on utterances distorted with white noise (Figure 10, Figure 11 and Figure 12), we can derive the following rules:

- If the length of utterance is short and signal-to-noise ratio is very low (i.e., SNR range from −5 to 5), then the weight of GMM-MFCC MLC is high and the weight of GMM-GFCC MLC is very high and the weight of GMM-MFCC supervector SVM is very low and the weight of GMM-GFCC supervector SVM is low.
- If the length of utterance is medium and signal-to-noise ratio is very low (i.e., SNR range from 5 to 15), then the weight of GMM-MFCC MLC is very high and the weight of GMM-GFCC MLC is low and the weight of GMM-MFCC supervector SVM is high and the weight of GMM-GFCC supervector SVM is very low.
- If the length of utterance is short and signal-to-noise ratio is low (i.e., SNR range from 5 to 15), then the weight of GMM-MFCC MLC is low and the weight of GMM-GFCC MLC is very high and the weight of GMM-MFCC supervector SVM is very low and the weight of GMM-GFCC supervector SVM is high.

Considering five membership functions for input 2 (signal-to-noise ratio) and three membership functions for input 1 (length of utterance), 15 IF-THEN rules are derived for each type of noise. One of the shortcomings of this approach is that for each type of noise, a set of IF-THEN fuzzy rules need to be derived.

## 4. Experimental Results

**Figure 19:**
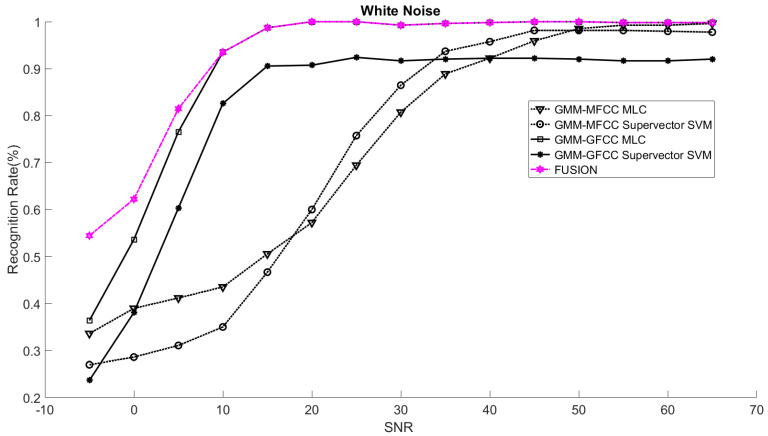
Rank-1 recognition rates of the fusion system and the base classifiers (two-stage) on utterances distorted with white noise.

**Figure 20:**
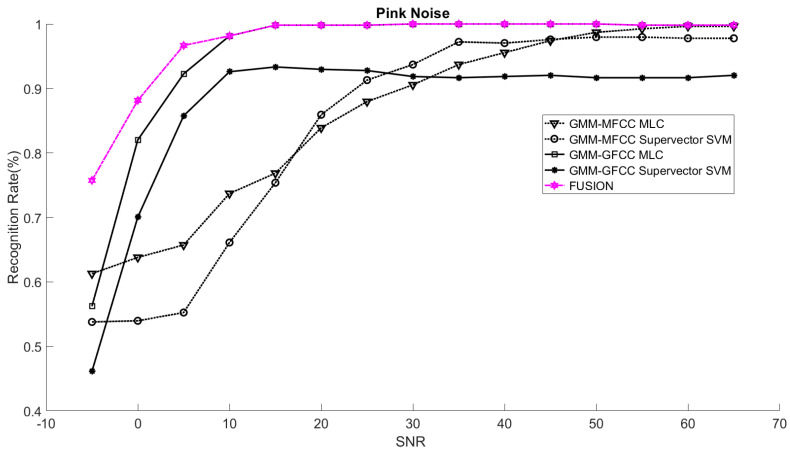
Rank-1 recognition rates of the fusion system and the base classifiers (two-stage) on utterances distorted with pink noise.

**Figure 21:**
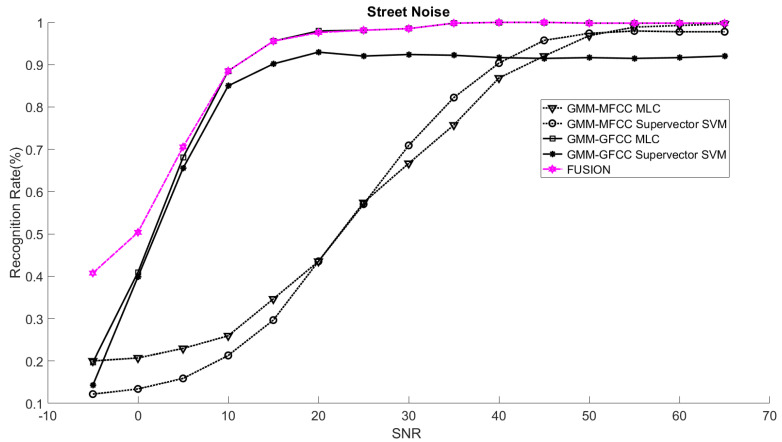
Rank-1 recognition rates of the fusion system and the base classifiers (two-stage) on utterances distorted with street noise.

The TIDIGITS database was used in this research study. TIDIGITS is a speech dataset which was originally collected at Texas Instruments, Inc., Dallas, TX, USA. The corpus was collected in a quiet environment and digitized at 20 kHz. The TIDIGITS corpus contains 326 speakers categorized into four groups (111 men, 114 women, 50 boys and 51 girls) each pronouncing 77 digit sequences. Only men and women groups were used in the experiments. Speech signals of 40 speakers (20 males and 20 females) out of 225 were randomly chosen in this study. The database was selected to emphasize that the proposed system was trained on limited speech data and that is not necessary to have phonetically rich utterances and at the same time is easy to pronounce. The main point is to facilitate collection of a similar database in social robot environments, such as robots working with speech impairment person or robots working with people with developmental disability as well as robots working in assisted living and hospitals [35,63,64]. Additionally, the choice of the number of speakers was selected to emulate social human–robot interaction tasks where social robots interact with a limited number of persons [65]. The speech samples were divided equally into three sets; namely training, development, and testing sets. The training set was used for speaker modeling and training base classifiers while the development set was used to study the recognition rate as a function of SNR and length of utterance. Consequently, we derived the relevant IF-THEN rules. The testing set was used to test the proposed systems under different noisy conditions. The testing set was composed of unseen data, not used in the development sample set of the system. It is worth noting that the training set was composed of clean speech signals while the speech signals in development and testing set were distorted with different noises at different SNR levels. At the test time, prior knowledge about the type of noise is assumed. The CASA-based approach presented in [66] was adopted to estimate the SNR of speech signal. The performance of the proposed speaker identification system was evaluated on three different noises (white, pink, street) at range of SNR (−5–65 dB). The overall recognition rates of the proposed system (fusion) and the recognition rates of the base classifiers were computed for the aforementioned types of noise. The rank-1 recognition rates of the fusion system were plotted as a function of SNR as shown in Figure 19, Figure 20 and Figure 21. Additionally, the rank-1 recognition rates of the base classifiers when they are used within the proposed system (two-stage) are depicted in the same figures. The performance of the base classifiers when they are used within the proposed system were compared with that of the same base classifiers when they are used independently without first stage classifier (i.e., without gender classification) [19,27,67] as shown in Table 1, Table 2 and Table 3.

### Discussion

The results suggest that the recognition rates of the base classifiers that are used within the proposed architecture (i.e., exploiting the gender information in the first-stage classifier) outperform that of the same classifiers when they are used independently (without the first-stage classifier). However, the recognition rates of these base classifiers and consequently the overall recognition rate of the proposed system is highly affected by the performance of the first classifier (gender classification). Additionally, the outcome of fusing all the base classifiers within the proposed architecture outperforms the performance of the best of the base classifiers at low SNR and matches the performance of the best of the base classifiers at high SNR. The proposed fusion system exploits the knowledge about the strengths and the weaknesses of the base classifiers in order to improve the overall performance of the system. The knowledge about the strengths and the weaknesses of each base classifier at different combinations of SNR and length of utterance is used to increase the contribution of the strong classifier and to reduce the contribution of the weak classifier, consequently improving the overall performance of the system. For instance, when the speech signals are distorted with white noise, the GMM-GFCC classifier outperforms all other base classifiers at low SNR and short utterance. On the other hand, at low SNR and long utterance the GMM-MFCC MLC classifier is superior to all other base classifiers. Thus, more weights are given to these classifiers when they encounter similar conditions at test time. The weight factors are governed by fuzzy rules that are derived relying upon the performance of the base classifiers as discussed in Section 3.3. The proposed fusion approach considers the two base classifiers that were combined such that they complement each other (i.e., the selected base classifiers need to use different features or different models given priority to the base classifier that uses different feature vectors) whenever it is possible—in the light of their performance. For instance, considering street noise, the GMM-MFCC MLC classifier was selected to be combined with GMM-GFCC MLC classifier at low to medium SNR (approximately in the range of −3–12 dB) and short utterance even though GMM-GFCC supervector SVM has better performance than that of GMM-MFCC MLC classifier. However, the fuzzy inference system assigns most of the weight to GMM-GFCC MLC classifier as its performance is superior to the rest of the classifiers at this specific condition.

## 5. Conclusions

In the absence of a unique robust speaker identification system that demonstrates superior performance for applications where the system is expected to perform in challenging scenarios such as different types of environmental noise, at different levels of environmental noise (low SNR to high SNR), and with only access to short utterances (at test time), the plausible contention is to integrate the advantages of using a multi-feature speaker recognition system with a multi-classifier speaker recognition system. In this study, two types of speech-based features (short-term spectral and prosodic features) and three powerful classifier systems (SVM, GMM, and GMM supervector-based SVM classifiers) are incorporated within an elegant architecture to identify the speaker and his/her gender as a byproduct. Exploiting prosodic features to cluster the population into two groups reduces the population size and builds a strong coupling between the speaker-dependent model and the UBM. The reduction in population size as well as deriving the speaker-dependent model from the gender-dependent model, improves the recognition rates of the base classifiers and reduces the computational cost. The recognition rates of the base classifiers (namely GMM-MFCC MLC, GMM-GFCC MLC, GMM-MFCC supervector SVM, and GMM-GFCC supervector SVM), were improved by maximum (64.62%, 141.62%, 56.85%, and 119.35%), (35.23%, 41.05%, 31.49%, and 77.85%), and (52.32%, 103.60%, 33.33%, and 105%) when evaluated on short utterances distorted with white noise, pink noise, and street noise, respectively. Moreover, combining the base classifiers at score level by assigning weights proportional to their performance at different conditions (combinations of SNR and length of utterance), improve the overall recognition rate of the proposed speaker recognition system particularly at low SNR and short utterance.

## Figures and Tables

**Table 1:** Comparison of recognition rates for the base classifiers when they are used within the proposed system (blue) with that of the same base classifiers when they are used independently (red) on utterances distorted with white noise.

SNR (dB)	Success Recognition Rate of Base Classifiers (%)											
	GMM-MFCC MLC			GMM-MFCC Supervector SVM			GMM-GFCC MLC			GMM-GFCC Supervector SVM		
	Indep.	Two -Stage	|%Δ|	Indep.	Two -Stage	|%Δ|	Indep.	Two -Stage	|%Δ|	Indep.	Two -Stage	|%Δ|
−5	22.63	33.6	48.46	20.37	26.98	32.48	15.06	36.38	141.62	10.79	23.67	119.35
0	24.57	38.96	58.55	20.19	28.59	41.59	35.74	53.61	50	22.52	38.06	69.05
5	25.01	41.17	64.62	20.76	31.06	49.57	54.08	76.56	41.57	45.32	60.31	33.07
10	26.94	43.52	61.51	22.31	35.00	56.85	74.35	93.52	25.78	71.67	82.59	15.25
15	35.74	50.56	41.45	31.2	46.67	49.55	85.93	98.70	14.87	84.26	90.56	7.47
20	44.44	57.22	28.75	50.56	60.00	18.68	91.94	100	8.76	88.8	90.74	2.19
25	58.06	69.44	19.62	67.22	75.74	12.67	96.67	100	3.45	91.02	92.41	1.53
30	69.17	80.74	16.73	77.78	86.48	11.19	96.76	99.26	2.58	92.5	91.67	0.90
35	80.37	88.89	10.6	87.31	93.70	7.32	98.06	99.63	1.61	92.04	92.04	0.0
40	85.56	92.22	7.79	93.24	95.74	2.68	98.8	99.81	1.03	91.11	92.22	1.22
45	91.39	95.93	4.96	95.56	98.15	2.71	99.17	100	0.84	91.3	92.22	1.01
50	94.72	98.52	4.01	96.67	98.15	1.53	99.35	100	0.65	91.48	92.04	0.61
55	96.39	99.26	2.98	97.41	98.15	0.76	99.54	99.81	0.28	91.76	91.67	0.1
60	96.94	99.26	2.39	97.5	97.96	0.47	99.44	99.81	0.37	91.85	91.67	0.2
65	97.41	99.63	2.28	97.5	97.78	0.28	99.54	99.81	0.28	91.67	92.04	0.4

**Table 2:** Comparison of recognition rates for the base classifiers when they are used within the proposed system (blue) with that of the same base classifiers when they are used independently (red) on utterances distorted with pink noise.

SNR (dB)	Success Recognition Rate of Base Classifiers (%)											
	GMM-MFCC MLC			GMM-MFCC Supervector SVM			GMM-GFCC MLC			GMM-GFCC Supervector SVM		
	Indep.	Two -Stage	|%Δ|	Indep.	Two -Stage	|%Δ|	Indep.	Two -Stage	|%Δ|	Indep.	Two -Stage	|%Δ|
−5	46.22	61.27	32.58	40.91	53.79	31.49	39.86	56.23	41.05	25.94	46.13	77.85
0	47.18	63.80	35.23	41.46	53.97	30.17	68.89	82.03	19.07	56.47	70.05	24.05
5	50.74	65.72	29.54	44.87	55.25	23.14	84.23	92.26	9.54	75.38	85.76	13.77
10	56.94	73.70	29.43	53.15	66.11	24.39	93.06	98.15	5.47	88.15	92.59	5.04
15	65.56	76.85	17.23	65.09	75.37	15.79	97.31	99.81	2.57	91.76	93.33	1.72
20	73.06	83.89	14.83	78.33	85.93	9.69	98.89	99.81	0.94	92.87	92.96	0.1
25	79.07	87.96	11.24	86.11	91.30	6.02	99.07	99.81	0.75	93.24	92.78	0.5
30	83.06	90.56	9.03	91.67	93.70	2.22	99.54	100	0.47	93.15	91.85	1.39
35	87.04	93.70	7.66	93.61	97.22	3.86	99.63	100	0.37	92.13	91.67	0.5
40	91.02	95.56	4.98	95.19	97.04	1.95	99.54	100	0.47	91.57	91.85	0.3
45	94.81	97.41	2.73	96.20	97.59	1.44	99.44	100	0.56	91.30	92.04	0.81
50	96.20	98.70	2.6	96.67	97.96	1.34	99.54	100	0.47	91.67	91.67	0.0
55	97.04	99.26	2.29	97.13	97.96	0.86	99.44	99.81	0.37	91.85	91.67	0.2
60	97.04	99.63	2.67	97.22	97.78	0.57	99.54	99.81	0.28	91.76	91.67	0.1
65	97.41	99.63	2.28	97.50	97.78	0.28	99.54	99.81	0.28	91.67	92.04	0.4

**Table 3:** Comparison of recognition rates for the base classifiers when they are used within the proposed system (blue) with that of the same base classifiers when they are used independently (red) on utterances distorted with street noise.

SNR (dB)	Success Recognition Rate of Base Classifiers (%)											
	GMM-MFCC MLC			GMM-MFCC Supervector SVM			GMM-GFCC MLC			GMM-GFCC Supervector SVM		
	Indep.	Two -Stage	|%Δ|	Indep.	Two -Stage	|%Δ|	Indep.	Two -Stage	|%Δ|	Indep.	Two -Stage	|%Δ|
−5	13.14	20.02	52.32	9.14	12.19	33.33	9.66	19.67	103.6	6.96	14.27	105
0	13.76	20.73	50.65	10.45	13.4	28.21	34.4	40.92	18.96	27.07	39.85	47.19
5	16.79	22.93	36.56	13.27	15.89	19.73	63.56	68.07	7.1	57.6	65.54	13.79
10	22.31	25.93	16.18	17.87	21.30	19.17	81.48	88.52	8.64	78.8	85	7.87
15	31.57	34.63	9.68	28.43	29.63	4.23	89.81	95.56	6.39	87.96	90.19	2.53
20	44.63	43.52	2.49	42.22	43.52	3.07	93.43	97.96	4.86	91.67	92.96	1.41
25	56.76	57.41	1.14	55.19	57.04	3.36	96.3	98.15	1.92	92.5	92.04	0.5
30	66.85	66.67	0.28	68.89	70.93	2.96	98.43	98.52	0.09	92.5	92.41	0.1
35	74.72	75.74	1.36	81.67	82.22	0.68	99.44	99.81	0.37	92.69	92.22	0.5
40	82.31	86.85	5.51	87.31	90.37	3.5	100	100	0	91.85	91.67	0.2
45	89.26	92.04	3.11	92.31	95.74	3.71	99.72	100	0.28	91.76	91.48	0.3
50	94.17	96.85	2.85	95.93	97.41	1.54	99.63	99.81	0.19	91.94	91.67	0.3
55	96.39	98.89	2.59	97.04	97.96	0.95	99.63	99.81	0.19	91.85	91.48	0.4
60	97.04	99.26	2.29	97.22	97.78	0.57	99.54	99.81	0.28	91.67	91.67	0
65	97.41	99.63	2.28	97.5	97.78	0.28	99.54	99.81	0.28	91.67	92.04	0.4

## Data Availability

The data presented in this study are available on request from the corresponding author.

## Appendix A

**Table A1:** Prosodic feature vector.

Name	Statistic Measure
Fundamental frequency (F0)	Median, Max, and Min.
Spectral centroid (SC)	Mean and Std.
Spectral flatness measure (SFM)	Mean and Std.
Shannon entropy (SE)	Mean and Std.
Harmonics-to-noise ratio (HNR)	Mean and Std.
Jitter	Median
Shimmer	Median
The first three formant (F1,F2,F3)	Median
